# Discography aids definitive diagnosis of posterior epidural migration of lumbar disc fragments: case report and literature review

**DOI:** 10.1186/s12891-017-1516-2

**Published:** 2017-04-11

**Authors:** Morito Takano, Tomohiro Hikata, Soraya Nishimura, Michihiro Kamata

**Affiliations:** 1 Department of Orthopaedic Surgery, Spine Center, Kitasato Institute Hospital, 5-9-1 Shirokane, Minato-ku, Tokyo 108-8642 Japan; 2 Department of Orthopaedic Surgery, Keiyu Hospital, 3-7-3 Minatomirai, Nishi-ku, Yokohama, Kanagawa 220-8521 Japan

**Keywords:** Lumbar disc herniation, Discography, Posterior epidural migration of lumbar disc fragments

## Abstract

**Background:**

Posterior epidural migration of lumbar disc fragments (PEMLDF) is extremely rare. It is often confused with other posterior lesions and is usually diagnosed intraoperatively. We here describe the use of preoperative discography in the diagnosis of PEMLDF.

**Case presentation:**

A 78-year-old man presented with acute low back pain, gait disturbance, and paresthesia in both legs. Magnetic resonance imaging showed a mass located posteriorly and laterally to the left aspect of the dural sac at the L3 level. The initial diagnosis indicated PEMLDF, malignancy, spontaneous hematoma, or epidural abscess. L3/4 discography clearly showed leakage of the contrast medium into the posterior dural space, indicating PEMLDF. The lesion was identified intraoperatively as a herniated-disc fragment, consistent with the preoperative discography.

**Conclusion:**

PEMDLF is difficult to diagnose preoperatively. Discography is useful for the definitive diagnosis of PEMDLF prior to surgery.

## Background

When lumbar discs herniate, the nucleus pulposus usually herniates posteriorly or posterolaterally. Lombardi first reported the posterior epidural migration of lumbar disc fragments (PEMLDF) as a rare case in 1973 [1]. PEMLDF is often confused with other lesions that form in the posterior epidural space, such as malignancy, hematoma, and abscesses [2–4], making it difficult to make a definitive diagnosis of PEMLDF prior to surgery. In all reported cases of PEMDLF, magnetic resonance imaging (MRI) at the first visit showed posterior epidural lesions [5]. We here report a case of PEMLDF diagnosed by discography prior to surgery, and discuss the use of discography in the diagnosis of PEMLDF.

## Case presentation

**Fig. 1:**
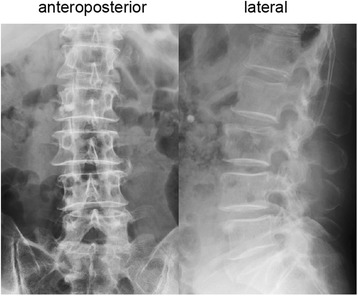
Preoperative radiographs. These are the anteroposterior and the lateral views of the lumbar spine. The preoperative radiographs showed slight wedging deformity and degenerative disc changes

**Fig. 2:**
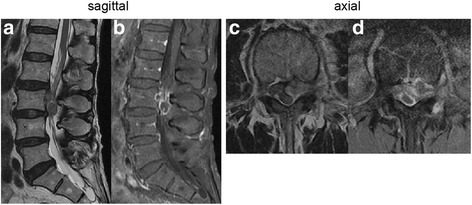
Preoperative MRI of the lumbar spine without and with intravenous contrast. T2-weighted MRI in the sagittal and axial planes showed a posterior epidural mass extending from the left past the midline to the contralateral posterior side (**a** and **c**). Fat-suppressed T1-weighted MRI in the sagittal and axial planes showed a lesion with a heterogeneous ring-like enhancement at the L3 level and demonstrated a sequestered fragment with posterior and superior migration in the dural sac (**b** and **d**)

**Fig. 3:**
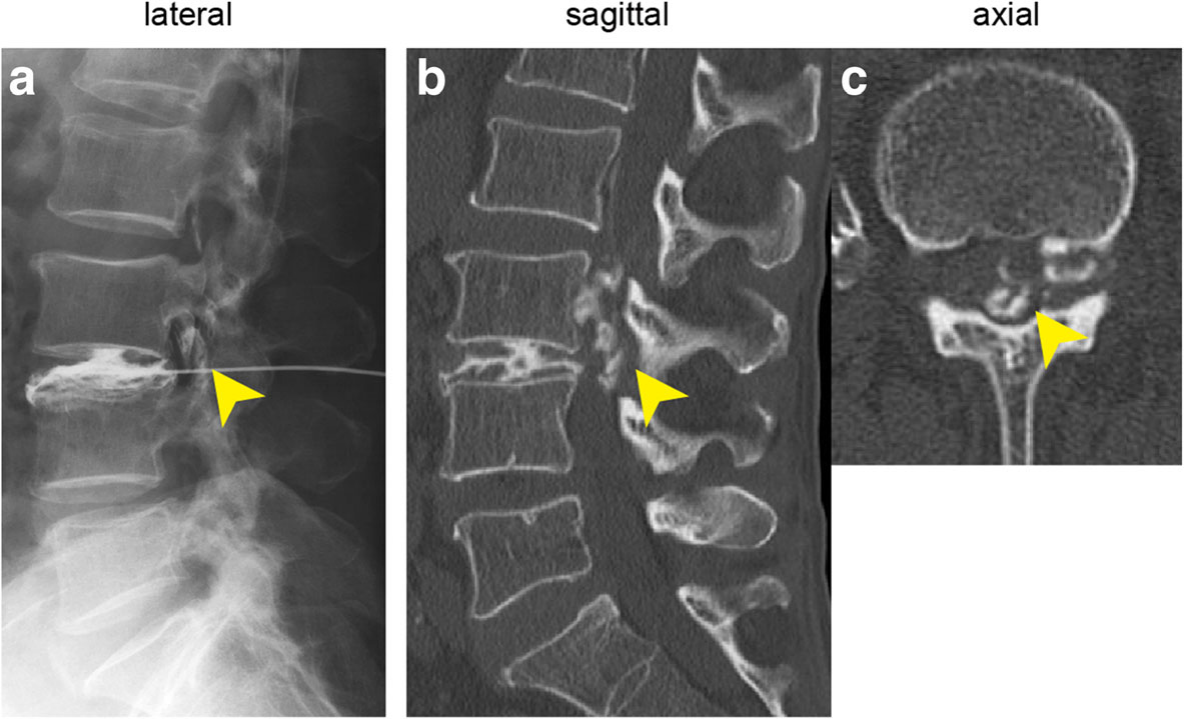
Discography and disco-computed tomography (CT). **a** Lateral view. (**b**, **c**) Sagittal and axial disco-CT view. Discography and disco-CT clearly revealed leakage of the contrast medium into the dorsal canal space from the L3/4 disc level (arrowheads)

**Fig. 4:**
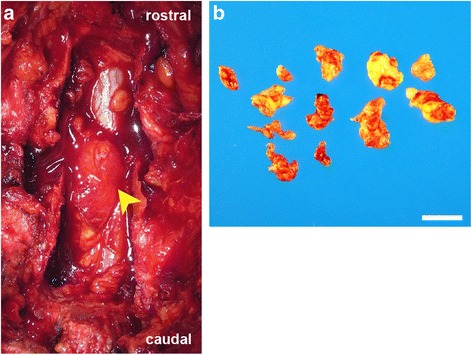
Gross appearance of the dorsal lumbar-disk herniation. **a** Intraoperative view of the dural sac after L2/3/4 laminectomy, showing the extracted disc fragment (arrowhead) and its attachment to the posterior dural sac. **b** The collected samples had a soft and yellowish appearance. Scale bar: 10 mm

**Fig. 5:**
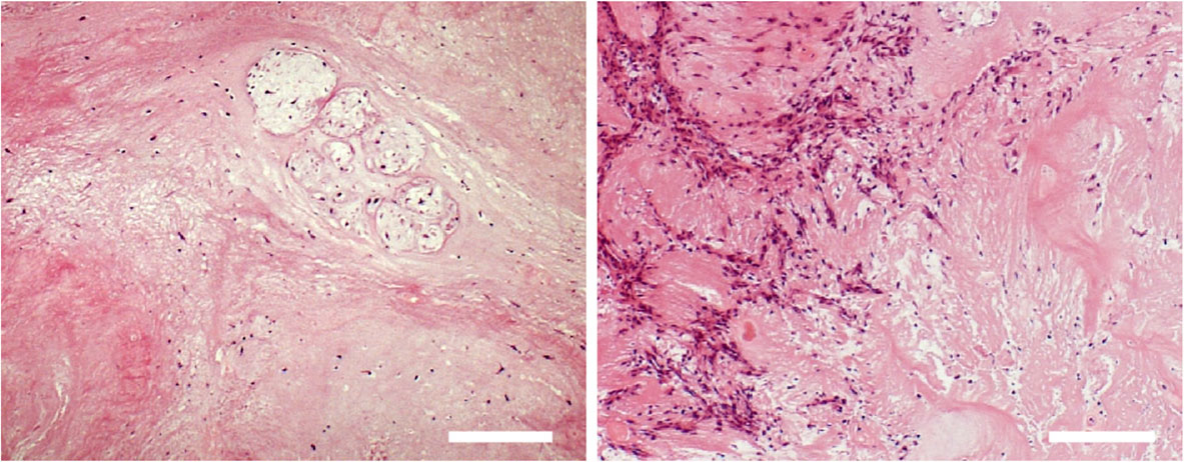
Histopathological findings. Representative images of HE-stained sections of the disc fragment extracted during the operation, showing liquefaction degeneration and granulation surrounding the sequestered disc fragment. Scale bar: 200 μm (hematoxylin and eosin [H & E] × 40)

A 78-year-old man developed acute low back pain, gait disturbance, and paresthesia in both legs; resting improved the paresthesia. Neurological examination revealed weakness with a Manual Muscle Testing (MMT) grade of 3 in the extensor hallucis longus and peroneus longus muscles of both legs. Bladder and bowel function were normal. He has had no past history and taken no medicine such as antiplatelet. His blood analysis showed C-reactive protein and complete blood count with differential were within normal limits, making an infectious process unlikely. Plain radiographs of the lumbar spine showed degenerative changes (Fig. 1). MRI showed a mass located posteriorly and laterally to the left aspect of the dural sac at the L3 level, and contrast-enhanced MRI showed a lesion with a heterogeneous ring-like enhancement at the L3 level (Fig. 2). We performed discography and disco-computed tomography (CT) to diagnose the dorsal epidural pathologies, expecting that the L3/4 discography would support a definitive diagnosis of lumbar disc herniation. Discography of the intervertebral disc at L3/4 level was performed using a standard posterolateral approach with a Hijikata discographic threehold needle (Tanaka Medical Instruments, Tokyo, Japan). For discography, the needle was inserted into the center of the disc under fluoroscopic control. Omnipaque 240 (3.0 mL; Daiichi Sankyo Company, Tokyo, Japan) was injected into the disc until contrast medium leaked out of the disc into the outside. After the injections were complete, the CT scan was performed to further analyze the L3/4 injected disc. However, the L3/4 discography and disco-CT clearly showed leakage of the contrast medium into the posterior dural space (Fig. 3). Based on these findings, we diagnosed neurological deficit due to PEMLDF. We performed an L2/3/4 laminectomy and separated a 30-mm-wide mass, embedded in fibrous epidural tissue, from the posterolateral aspect of the dural sac (Fig. 4a). No adhesion was seen. The huge disc fragment had been extruded into the epidural space and wrapped around from the outside of the nerve root. After removing the mass piece by piece (Fig. 4b), we detected a rupture in the left side of the L3-4 disc at the posterior longitudinal ligament. The compression on dura and nerve roots was totally removed. Consistent with observations during the operation, histopathological analysis revealed liquefaction degeneration and granulation surrounding the sequestered disc fragment (Fig. 5). The patient’s symptoms improved immediately after surgery. He recovered motor function to MMT 4–5 in both legs and was able to walk more smoothly. He identified the strength of the lower extremities improving and recovered fully after 3 months.

## Discussion

PEMLDF occurs infrequently: only 75 cases, including the present case, are reported in the English literature [5]. PEMLDF occurs in middle-aged persons (mean age 53.11 years), possibly owing to the dynamics of spinal degeneration with aging [6–9]. PEMLDF commonly occurs at the upper lumbar levels, especially at the L3/4 level (39.2%). Disc fragments most commonly migrate along a posterior and posterolateral route to the frontal epidural space; the second most common migration corridors are the cranial or caudal (with equal frequency) [10, 11]. Some authors suggest that disc fragments rarely migrate into the posterior epidural region because it requires passing through anatomical barriers such as the posterior longitudinal ligament, peridural or lateral membrane, epidural venous plexus, epidural fat, nerve root, and dura [12–14].

Clinically, PEMLDF can present with symptoms ranging from low back pain to radiculopathy and cauda equine syndrome [15]. Although MRI remains the modality of choice for investigating spinal disorders, PEMLDF can be challenging to diagnose since this type of herniation resembles other epidural pathologies such as abscess, acute hematoma, and malignancy [9, 12, 16, 17]. Malignancy usually produces a solid and rather homogeneous postcontrast enhancement. On the other hand, abscess and hematoma usually have a peripheral rim of enhancement and are associated with an infectious illness, a history of trauma, or antiplatelet drugs.

MRI is the useful imaging tool for the diagnosis of PEMLDF. It appears isointense with the intervertebral disc on T1-weighted images. On the other hands, T2-weighted images reveal a variable intensity of the lesion, with about 80% of all lesions appearing hypointense and the remaining 20% being isointense [6]. On contrast-enhanced MRI images, it appears like a cyst with enhanced rims. However, the MRI appearances of PEMLDF are not specific and they are also found in other posterior epidural lesions. Thus, a definitive diagnosis can at times be made intraoperatively.

Matsumoto et al. used discography and disco-CT to make a definitive diagnosis of intradural lumbar disc herniation [18], and noted that the contrast medium was not contained in the disc during discography, but also spread intrathecally in a pattern resembling a myelogram. In the present case, clinical history and enhanced MRI findings led us to suspect PEMLDF, and we used discography and disco-CT to make a definitive diagnosis. By performing the discography and disco-CT, we could distinguish PEMLDF from other spinal pathologies, such as malignancy, spontaneous hematoma, or epidural abscess. Furthermore, the discography and disco-CT allowed us to distinguish the L3/4 lumbar-disc herniation from other segments and to understand the whole aspect of the PEMLDF.

## Conclusion

Our report shows that discography is useful for making a definitive preoperative diagnosis of PEMLDF. Our case suggests that discography and disco-CT should be performed when the clinical history and enhanced MRI findings suggest the presence of PEMLDF.

## Abbreviations

CT: Computed tomography
MMT: Manual Muscle Testing
MRI: Magnetic resonance imaging
PEMLDF: Posterior epidural migration of lumbar disc fragment
